# NTDs in the 2020s: An epic struggle of effective control tools versus the Anthropocene

**DOI:** 10.1371/journal.pntd.0007872

**Published:** 2020-09-24

**Authors:** Peter J. Hotez

**Affiliations:** 1 Departments of Pediatrics and Molecular Virology & Microbiology, Texas Children’s Hospital Center for Vaccine Development, National School of Tropical Medicine, Baylor College of Medicine, Houston, Texas, United States of America; 2 Hagler Institute for Advanced Study at Texas A&M University, College Station, Texas, United States of America; 3 Department of Biology, Baylor University, Waco, Texas, United States of America; 4 James A Baker III Institute of Public Policy, Rice University, Houston, Texas, United States of America; 5 Scowcroft Institute of International Affairs, Bush School of Government and Public Service, Texas A&M University, College Station, Texas, United States of America; Weill Cornell Medical College, UNITED STATES

The 2000s and 2010s saw tremendous strides in the global control of neglected tropical diseases (NTDs) through a combination of mass drug administration (MDA), enhanced vector control, and, lately, the development and introduction of new vaccines. However, modern forces linked to the Anthropocene epic, including conflict, political instability, and climate change, may offset these public health gains. Therefore, progress in controlling NTDs in the coming decade may depend on our ability to overcome or circumvent the Anthropocene—our newest geological epoch caused by human activity—hurdles of the 2020s.

## The modern framework of NTDs: Mass treatment and vector control

In the years following the launch of the Millennium Development Goals in 2000, a group of scientists and experts committed to parasitic disease control proposed the expansion of preventive chemotherapy MDA activities, while simultaneously integrating MDA by combining treatment interventions through the administration of a package of essential medicines [1]. The scale-up and integration of MDA heralded a new era of NTDs, to the point that more than one billion people now receive annual access to essential medicines for three soil-transmitted helminth infections, schistosomiasis, lymphatic filariasis (LF), and onchocerciasis, as well as blinding trachoma, an important bacterial infection [2].

As 2020 approaches, we will enter our third decade of integrated mass treatment with some impressive results. According to the World Health Organization (WHO), approximately 600 million children were treated for soil-transmitted helminth infections in 2017 (the most recent year of WHO reporting), representing almost 70% of children requiring treatment, while 100 million children and adults were treated for schistosomiasis—almost one-half of those requiring treatment [3]. For trachoma, almost 90 million people received azithromycin or other antibiotics in 2018, and now, eight nations have achieved elimination status, with five additional countries expected to attain that goal [4]. For LF, almost 500 million people received MDA in 2017, representing more than one-half of the global population requiring treatment [5]. Moreover, about 550 million people no longer require MDA because LF was eliminated in multiple areas [5]. Approximately 70% of the 200 million people requiring MDA for onchocerciasis now receive mass treatment, while 1.8 million people living in multiple countries no longer require treatment due to successful elimination efforts [6]. Thus, through scale-up and integration of MDA, we can envision the eventual elimination of trachoma, LF, and onchocerciasis. Still another observation relevant to MDA is that scale-up and mass treatment have also yielded important collateral public health benefits, including reductions in the global prevalence of yaws, scabies, and other soil-transmitted helminthiases (such as oesophagostomiasis) and even overall reductions in child mortality [7].

Outside of MDA, through different approaches, we are making progress towards eliminating other NTDs. Dracunculiasis is near elimination, with multiple human cases reported only from Chad and South Sudan [8]. Through surveillance, case detection, and treatment, together with vector control, there are now fewer than 10,000 reported cases of human African trypanosomiasis, with the Democratic Republic of Congo (DR Congo) accounting for more than 80% of the cases [9, 10]. Through MDA, we are also making progress in leprosy control. WHO notes that over the past four years, there has been an annual 5% decrease in the number of new leprosy cases [11]. Many of these elimination targets are being pursued under the auspices of a 2010 London Declaration on NTDs [12]. In partnerships with communities, new vector control technologies, including genetically-modified mosquitoes and other arthropods, will also produce an important global health impact [13].

## New vaccines

As global mass-treatment efforts continue, new vaccines are being advanced with the hope that they could become licensed and integrated into MDA or childhood vaccination programs in the 2020s. Anthelminthic vaccines targeting schistosomiasis and hookworm infection are advancing in clinical trials, while a new onchocerciasis vaccine is completing preclinical development, with the hope that these new and innovative technologies might accelerate global control or elimination [14–17]. New leishmaniasis vaccines are being developed, possibly for use in the Middle East or East Africa where the disease burden is the highest [18], as is a new therapeutic Chagas disease vaccine to enhance efficacy of antiparasitic drug treatment [19]. New arbovirus vaccines for dengue, Zika, and chikungunya are also in various stages of clinical testing, and at least one dengue vaccine has already been licensed [20]. A new Ebola virus vaccine is being used to combat a large epidemic in eastern DR Congo, with more than 200,000 people vaccinated to date and preliminary assessments suggesting it is highly effective [21]. Similarly, cholera vaccines have been stockpiled by WHO and have found use in multiple reactive settings where outbreaks are underway [22]. Many of the vaccines that prevent or treat NTDs are only partially protective so they may not necessarily replace existing control approaches, but rather they would likely be developed and implemented as companion technologies [23].

## The Anthropocene counterpunch

In a perfect world, the continued scale-up of mass treatment, together with the eventual integration of NTD vaccines, would lead to the global elimination of the world’s NTDs. But not so fast: We do not yet have in place a global financing mechanism to support the advanced clinical development and licensure of the most urgently needed NTD vaccines, and, unless we can figure this out, many of these vaccines may never be produced [23]. Another major hurdle is the stark reality that in many of the poorest nations, health systems remain profoundly depleted, especially in some of the largest sub-Saharan African nations (such as DR Congo and Nigeria), where it is anticipated that 40% of the world’s extremely poor people will live by the year 2050 [24]. A concern is that such nations might experience difficulties incorporating new and appropriate technologies.

**Fig 1:**
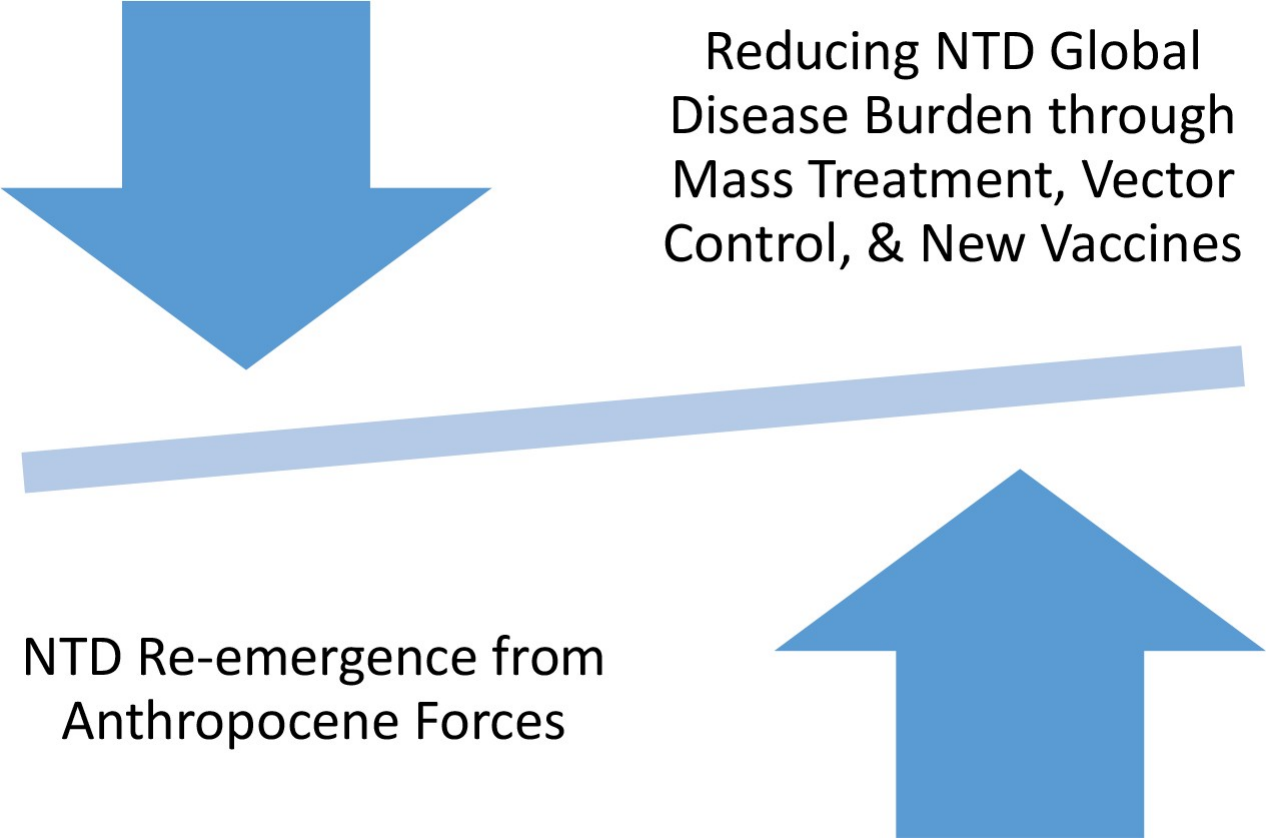
Opposing forces of new public health tools versus modern Anthropocene forces. NTD, neglected tropical disease.

A picture is emerging in which the greatest hurdle that might thwart our ability to fully realize the benefits of mass treatment, enhanced vector control, and new vaccines is the constellation of social and physical determinants sometimes known as the Anthropocene (Fig 1) [25]. The Anthropocene refers to our latest geological epic, which began when heightened human activity actually changed the physical and chemical composition of our planet. One of the best-known examples of Anthropocene forces is the increase in carbon dioxide and pollutants leading to climate change, but I also use this concept to embrace important social determinants including war, political collapse, and human migrations linked to refugee movements or urbanization, population growth, and deforestation. We recently reported how war and political instability is an especially potent driver and one linked to the highest prevalence of NTD rates globally [26].

Anthropocene forces will become a dominant theme as we advance towards the 2020s and ultimately could overwhelm our ability to control NTDs despite the promise of existing and future technologies and control tools. Anthropocene forces might even reverse our progress.

A good example of the struggle between NTD control tools and the Anthropocene is playing out now in DR Congo. In 2019, WHO declared a Public Health Emergency of International Concern (PHEIC) because of an expanding Ebola virus infection epidemic in eastern DR Congo [27]. In reality, Ebola was not the only catastrophic infection affecting this region, with some estimates indicating that more individuals were actually dying of measles than Ebola virus infection, while cholera had also emerged. An interesting point about all three infectious or neglected diseases is that highly effective vaccines were developed for each of these infections, together with the fact that the global health community put in place important efforts to ensure vaccine access. For example, the new recombinant vesicular stomatitis virus–Zaire Ebola virus (rVSV–ZEBOV) Ebola vaccine shows 90% or more protection against Ebola virus infection when used in programs of ring vaccination of patient contacts [28]. Despite the achievement of vaccinating more than 200,000 people with the new vaccine, the death toll from Ebola virus infection in eastern Congo continues to climb, thereby necessitating WHO to issue the PHEIC.

A major reason is the inability of vaccinators to reach at-risk populations in conflict and politically unstable areas or, in some cases, outright violence to healthcare workers [28]. However, the Ebola virus situation in eastern Congo would be far worse without the vaccine, possibly as bad as what we saw in the 2013–2016 West African Ebola virus epidemic. The worsening situation there demonstrates the power of Anthropocene forces, particularly ongoing conflict and hostilities. In 2014, Daniel Bausch pointed out how a different set of Anthropocene forces, including climate change, deforestation, and urbanization, contributed to the West African epidemic [29].

## Concluding comments

I’m confident that the vaccinators and their support system, including Gavi, the Vaccine Alliance, WHO, and UNICEF, will overcome the current hurdles now hindering vaccination efforts against Ebola virus infection, measles, and cholera. However, we must recognize that Anthropocene forces now challenging NTD control are not restricted to DR Congo. Instead, they must become an important new theme for the 2020s, one the global health community must overcome or circumvent in order to achieve both Global Goals and London Declaration targets for the NTDs.
